# Astragaloside IV regulates autophagy-mediated proliferation and apoptosis in a rat model of PCOS by activating the PPARγ pathway

**DOI:** 10.22038/IJBMS.2022.64475.14179

**Published:** 2022-07

**Authors:** Mingxiao Wen, Wenjun Chen, Qun Zhou, Xiaoqing Dou

**Affiliations:** 1 Department of Gynaecology and Obstetrics, The First Affiliated Hospital of Zhejiang Chinese Medical University (Zhejiang Provincial Hospital of Traditional Chinese Medicine), Hangzhou 310000, China

**Keywords:** Apoptosis, Astragaloside IV, Autophagy, Polycystic ovary syndrome, PPARγ, Rat

## Abstract

**Objective(s):**

Astragaloside IV (AS-IV) is a bioactive saponin with a wide range of pharmacological effects. This study was aimed at investigating its potential effect on polycystic ovary syndrome (PCOS).

**Materials and Methods:**

Female Sprague-Dawley rats were randomly divided into five groups (control, PCOS, PCOS+AS-IV 20 mg/kg, PCOS+AS-IV 40 mg/kg, and PCOS+AS-IV 80 mg/kg). The pathological injury level of rat ovary was observed with hematoxylin-eosin (H&E) staining; enzyme-linked immunosorbent assay (ELISA) kit was utilized to measure the levels of luteinizing hormone (LH), follicle-stimulating hormone (FSH), and testosterone in rat serum; western blot detected autophagy-associated or peroxisome proliferator-activated receptor γ (PPARγ) pathway-related protein expression; immunofluorescence was performed to observe LC3 level in rat ovarian tissue. After co-treatment with AS-IV and PPARγ inhibitor, the proliferation in ovarian granulosa cell line KGN was examined employing cell counting kit-8 (CCK-8), EdU staining, and colony formation; cell apoptosis was observed with TdT-mediated dUTP nick-end labeling (TUNEL); apoptosis-related protein expression was assayed by western blot.

**Results:**

Treatment with AS-IV inhibited the ovarian pathological damage in PCOS rats. It also promoted the level of autophagy and activated PPARγ signaling in the rat PCOS model. In KGN cells, the level of autophagy and expression of PPARγ-related proteins were also elevated by AS-IV treatment. Furthermore, AS-IV facilitated autophagy, thus inhibiting KGN cell proliferation and promoting its apoptosis, through activating the PPARγ signaling pathway.

**Conclusion:**

AS-IV-activated PPARγ inhibits proliferation and promotes the apoptosis of ovarian granulosa cells, enhancing ovarian function in rats with PCOS.

## Introduction

Polycystic ovary syndrome (PCOS), characterized by reproductive dysfunction and abnormal glucose metabolism, is an endocrine disorder syndrome widely affecting females of reproductive age worldwide (1, 2). Patients with PCOS often present with symptoms, such as irregular menstrual cycles, increased weight, insulin resistance, excessive body hair, infertility, overproduction of androgens, and polycystic ovarian morphology (3-5). These symptoms and their severity may vary among individuals, and the pathophysiology of the condition is not yet fully understood. According to various diagnostic criteria based on the complexity of the symptoms, the incidence of PCOS worldwide ranges from 6% to almost 18% (6, 7). Even though PCOS itself may not be a critical condition, it is considered to be associated with a relatively higher risk of developing other diseases, including type 2 diabetes mellitus, cardiovascular disease, and endometrial cancer (8-10). Clinically applied treatment for PCOS at present is mostly limited to pharmacological therapies targeting anovulatory infertility, menstrual disturbances, or hyperandrogenism, involving clomifene, metformin, and oral contraceptive pills (11). These strategies may be ideal in terms of alleviating the symptoms temporarily; however, an effective approach for comprehensive improvement of ovarian function, particularly polycystic ovarian morphology, remains a clinical challenge and an interest of researchers.

Astragaloside IV (AS-IV) is a highly purified compound derived from *Astragalus membranaceus *(Huangqi), a Chinese herbal plant with anti-oxidant, anti-inflammatory, anti-bacterial, and anti-atherosclerotic properties that has been extensively used in traditional Chinese medicine for more than 20 centuries (12, 13). AS-IV has also proven to exert potent pharmacological effects on cardiac function, the endocrine system, and liver health, amongst other benefits. (14, 15). Recent studies have revealed the autophagy-regulating effects of AS-IV, through which it inhibits the epithelial-mesenchymal transition of podocytes in glomerular diseases and improves the high glucose-induced morphological changes of cardiomyocytes in diabetic cardiomyopathy (16, 17). Autophagy plays a vital role in the development of PCOS. Its aberrant level in granulosa cells is related to insulin resistance and luteal degeneration (18, 19). Therefore, it was hypothesized that AS-IV may regulate autophagy in PCOS and thus improve ovarian function. Treatment with AS-IV has been shown to augment peroxisome proliferator-activated receptor γ (PPARγ) signaling, thereby preventing memory impairment and hippocampal neuronal apoptosis (20). AS-IV as a natural PPARγ agonist inhibited the activity of (β-site amyloid precursor protein cleaving enzyme 1 (BACE1) and ultimately reduced the generation of β-amyloid (Aβ) (21). AS-IV suppressed neuroinflammation in mice via increasing PPARγ expression (22). AS-IV inhibited podocyte apoptosis by activating the PPARγ signaling pathway (23). Furthermore, activation of PPARγ can induce autophagy mediated by neuronally expressed developmentally down-regulated 4 (NEDD4) (24). PPARγ activation promoted NEDD4-mediated autophagy induction and Akt phosphorylation (25).

Therefore, the present study investigated the potential effects of AS-IV and its interaction with PPARγ on ovarian function *in vitro *and* in vivo*. We have speculated that AS-IV could improve the ovarian function of PCOS rats and suppress the proliferation and promote apoptosis of KGN cells by activating autophagy via the PPARγ pathway. 

## Materials and Methods

***Animals and rat model of PCOS***

A total of thirty female Sprague-Dawley (SD) rats (weighing 200–250 g; 8 weeks old) were purchased from the Experimental Animal Center, Shanghai Institute of Materia Medica Chinese Academy of Sciences. The rats were randomly divided into five groups (the control, PCOS, PCOS+AS-IV 20 mg/kg, PCOS+AS-IV 40 mg/kg, PCOS+AS-IV 80 mg/kg) and were group-housed in a temperature-constant environment (22 °C±2 °C, 60% humidity) under a 12-hr:12-hr light:dark cycle. Rats in the PCOS group (n=6) were subcutaneously injected with dehydroepiandrosterone (DHEA; Sigma-Aldrich; Merck KGaA) at a dose of 6 mg/kg/day for twenty days (26); those in the PCOS + AS-IV groups (n=18) were additionally injected with AS-IV (Shanghai Sunny Biotech Co, Ltd) at a dose of 20, 40, or 80 mg/kg/day for twenty days (27). During the experiment, rats were administered food and water *ad libitum*. The fasting glucose and fasting insulin levels were determined twenty days later, and the homeostasis model assessment of insulin resistance (HOMA-IR) was performed by referring to a reported method (28). These rats with HOMA-IR index >2.8 were considered the successful rat model of PCOS and could be used for further study. Finally, thirty rats were euthanized with pentobarbital sodium (165 mg/kg, IP) and the heartbeat was checked. The animal experiments in the present study were approved by the experimental Animal Management and Ethics Committee of Zhejiang University of Traditional Chinese Medicine and performed in accordance with the guidelines established by this committee.

***Estrous cycle***

During the experiment, a sterile cotton swab dipped in normal saline was slowly inserted into the vagina of the rats at 9 AM every day, and it was gently rotated twice in the vagina and evenly applied clockwise on the slide. After the vaginal smear, the cells were dried naturally and then stained with hematoxylin. Cell types and morphology were observed under a microscope and observed at the same time. The changes in vaginal diameter and hyperemia state of rats combined with the results of vaginal smear examination were used to judge the estrous cycle of rats.

***Cells, cell culture, and treatment***

The human ovarian granulosa cell line, KGN, was purchased from Guangzhou Cellcook Biotech Co., Ltd. The cells were cultured in DMEM/F-12 (no phenol red) (cat. no. CM2005; Guangzhou Cellcook Biotech Co., Ltd.) supplemented with 10% fetal bovine serum (Sbjbio Life Sciences). AS-IV at the concentrations of 20, 40, or 80 μg/ml was used to treat the cells for 48 hr. Cells pre-treated with the PPARγ inhibitor, GW9662, (20 μM; Selleck Chemicals) or the autophagy inhibitor, 3-methyladenine (3-MA; 1 mM; MedChem Express) were then treated with 80 μg/ml AS-IV to observe cell proliferation and apoptosis.

***Hematoxylin and eosin (H&E) staining***

Rat ovarian tissues were collected, fixed with 4% paraformaldehyde, and embedded in paraffin. After the tissues were sectioned into ~4 μm-thick sections, they were deparaffinized in xylol for 5 min, immersed in a gradient of ethanol solutions, and washed with distilled water for 2 min. The sections were then stained with hematoxylin followed by eosin (Wuhan Boster Biological Technology, Ltd.) for 5 and 2 min at room temperature, respectively. Finally, the sections were microscopically observed and photographed.

***Determination of serum levels of hormones and metabolic variables***

ELISA was performed using corresponding ELISA kits (Nanjing Jiancheng Bioengineering Institute) to detect the levels of insulin (cat. no. H203-1-1), glucose (cat. no. F006-1-1), luteinizing hormone (LH; cat. no. H206-1-2), follicle-stimulating hormone (FSH; cat. no. H101-1-2) and testosterone (cat. no. H090-1-1) in rat serum in accordance with the manufacturer’s protocols. HOMA-IR was calculated according to the following formula: plasma glucose (mmol/l) x serum insulin (mIU/l)/22.5.

***Western blot analysis***

Total protein was extracted from rat ovarian tissues or KGN cells using RIPA lysis butffer [Yeasen Biotechnology (Shanghai) Co., Ltd.]. The concentration of the protein was determined using a BCA kit [Yeasen Biotechnology (Shanghai) Co., Ltd.]. Total protein was then sufficiently denatured by boiling for 5 min in a water bath. 12% sodium dodecyl sulfate-polyacrylamide gel electrophoresis (SDS-PAGE) was performed to separate the protein (20 µg), which was subsequently transferred to a polyvinylidene fluoride (PVDF) membrane (Corning Inc.) and blocked with 5% skim milk at room temperature for 2 hr. The membrane was then incubated with one of the primary antibodies LC3I/II (#4108; dilution, 1:1000; Cell signaling technology), p62 (#39749; dilution, 1:1000; Cell signaling technology), Beclin1 (#3738; dilution, 1:1000; Cell signaling technology), PPARγ (ab272718; dilution, 1:1000; Abcam), Bcl-2 (ab196495; dilution, 1:1000; Abcam), Bax (ab32503; dilution, 1:1000; Abcam), cleaved-caspase3 (#9664; dilution, 1:1000; Cell signaling technology), cleaved-caspase9 (ab2324; dilution, 1:1000; Abcam), caspase3 (#9662; dilution, 1:1000; Cell signaling technology), caspase9 (ab32539; dilution, 1:1000; Abcam), and GAPDH (#5174; dilution, 1:1000; Cell signaling technology), followed by secondary antibody goat anti-rabbit IgG (ab205718; dilution, 1:2000; Abcam), for 2 hr at room temperature. Images of the proteins were developed using an enhanced chemiluminescence (ECL) kit (Beyotime Institute of Biotechnology).

***Immunofluorescence (IF) staining***

The level of light chain 3 (LC3) in rat ovarian tissues was assayed by IF staining. In brief, paraffin-embedded tissue sections were deparaffinized and hydrated followed by antibody retrieval. Following fixation and blockage, the sections were incubated with the primary antibody anti-LC3 (#4108; dilution, 1:400; Cell signaling technology) overnight at 4 °C, followed by incubation with the secondary antibody (#5174; dilution, 1:200; Cell signaling technology) for 60 min in the dark at room temperature. Finally, the sections were observed and photographed under an inverted fluorescence microscope (Olympus, Japan).

***Cell Counting Kit-8 (CCK-8) assay***

A CCK-8 kit (Beyotime Institute of Biotechnology) was used in this assay. KGN cells were inoculated into a 96-well plate with 100 μl culture medium per well and treated accordingly. After the cells were fully adherent to the wall, 10 μl CCK-8 solution was added to each well for 1 hr at 37 °C. The absorbance at 450 nm wavelength was determined following 2 hr of incubation.

***EdU staining***

Following cell culture and treatment in a 6-well plate, 10 μM pre-heated EdU working solution (Beyotime Institute of Biotechnology) was added to label the cells for 2 hr. Subsequently, 1 ml 4% paraformaldehyde was used to fix the cells for 15 min. The cells were then permeabilized by 0.3% Triton X-100 and incubated with 0.5 ml Click Additive Solution (Beyotime Institute of Biotechnology) for 30 min in the dark, followed by microscopic observation.

***Colony formation assay***

KGN cells in the logarithmic growth phase were digested with 0.25% trypsin (Hangzhou Putai Biotechnology Co., Ltd.), dissociated, and suspended in a complete culture medium. Cells in the density gradient of 50, 100, and 200 cells per dish were incubated with a 10 ml pre-heated culture medium and cultured in a humidified environment with 5% CO_2_ at 37 °C for 2 weeks. The culture was terminated when cell colonies could be observed macroscopically. The colonies were stained with Giemsa staining solution at room temperature for 15 min (Beyotime Institute of Biotechnology) before microscopic observation.

***TdT-mediated dUTP nick-end labeling (TUNEL)***

A TUNEL Apoptosis assay kit (Beyotime Institute of Biotechnology) was used in this assay. In brief, the cells were fixed by 4% paraformaldehyde, permeabilized with 0.3% Triton X-100 in PBS, and then blocked with 0.3% H_2_O_2_ in PBS. Following biotin labeling, streptavidin-HRP followed by DAB solution was added to develop the signals. Finally, cell apoptosis was observed and imaged using an inverted fluorescence microscope (Olympus, Japan).

***Statistical analysis***

Statistical analysis was performed using GraphPad Prism 6 software. Data are expressed as the mean ± standard deviation (SD). Group results were compared using one-way ANOVA followed by Tukey’s *post hoc* test. *P*<0.05 was considered to indicate a statistically significant difference. All experiments were independently conducted at least three times.

## Results

***AS-IV treatment improves ovarian injury, abnormalities of serum hormone levels, and the estrous cycle in PCOS rats***

**Figure 1:**
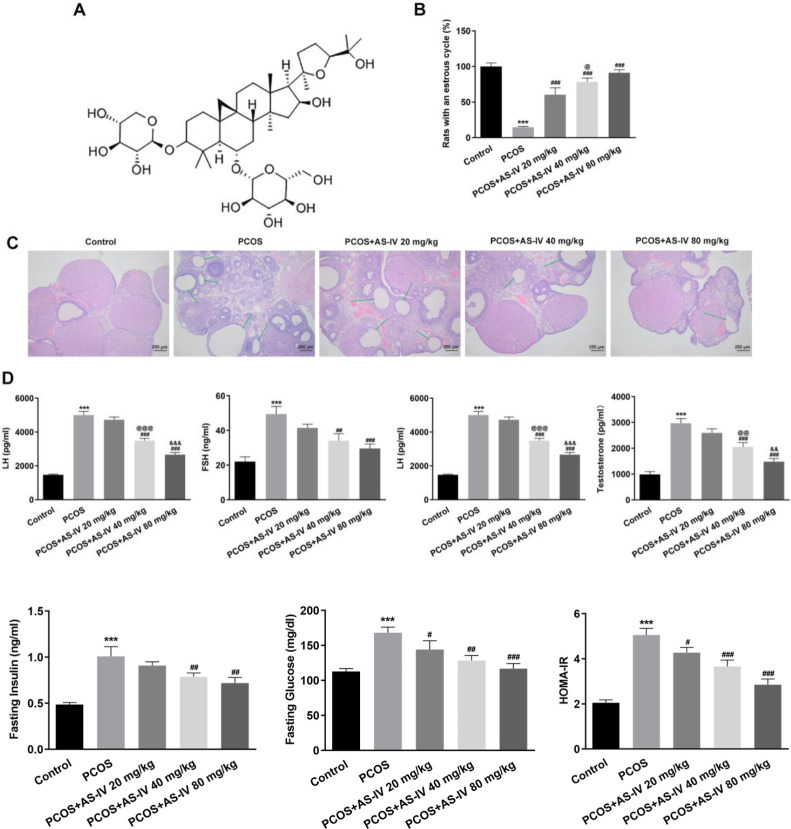
AS-IV treatment improves ovarian injury, abnormalities of serum hormone levels, and the estrous cycle in PCOS rats (A) Chemical formula of AS-IV. (B) The estrous cycle of rats before and after AS-IV treatment. (C) Pathological injury level of rat ovaries before and after AS-IV treatment observed through HE staining. (D-G) levels of LH, FASH, LH/FSH, and testosterone in rat serum before and after AS-IV treatment, detected by ELISA. Data are expressed as mean ± SD. ***P<0.001 versus Control. ##*P*<0.01, ###*P*<0.001 versus the PCOS group. @@*P*<0.01, @@@*P*<0.001 versus the +AS-IV 20 mg/kg group. &&P<0.01, &&&*P*<0.001 versus the +AS-IV 40 mg/kg group

AS-IV: Astragaloside IV; PCOS: Polycystic ovary syndrome; ELISA: Enzyme-linked immunosorbent assay

The pathological injury and ovarian function of PCOS rats after AS-IV treatment were analyzed. The chemical formula of AS-IV is illustrated in Figure 1A. The estrous cycle was used to estimate the ovarian function in PCOS rats (Figure 1B). All rats in the control group exhibited a normal estrous cycle. However, DHEA-induced PCOS rats showed a significantly impaired cycle; only 1 of 6 rats exhibited a normal estrous cycle in the PCOS group (compared with the Control group, *P*<0.001). In the presence of AS-IV at concentrations of 20, 40, and 80 mg/kg, the number of rats with a normal estrous cycle increased to 3, 4, and 5 of 6 rats in the PCOS group (compared with PCOS group, *P*<0.001; compared with +AS-IV 20 mg/kg group, *P*<0.05). The ovarian tissue of the rats with PCOS exhibited pathological injury, which was improved with the increasing concentration of AS-IV (Figure 1C). Furthermore, compared with the control group, the rats with PCOS exhibited an increased level of LH, FSH, and testosterone, as well as an increased LH/FSH value (compared with the Control group, *P*<0.001), which was reduced by AS-IV treatment in a dose-dependent manner (compared with PCOS group, *P*<0.01 and *P*<0.001; compared with +AS-IV 20 mg/kg group, *P*<0.01 and *P*<0.001; compared with +AS-IV 40 mg/kg group, *P*<0.01 and *P*<0.001) (Figures 1D-G). 

***AS-IV treatment improves insulin resistance in PCOS rats***

**Figure 2:**
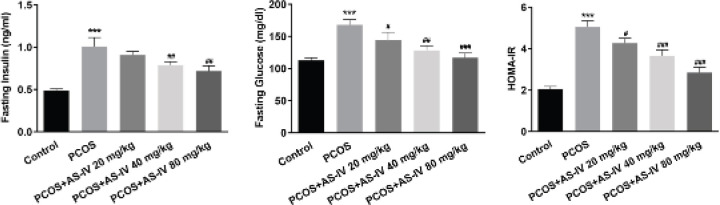
AS-IV treatment improves insulin resistance in PCOS rats. Levels of serum insulin, blood glucose and HOMA-IR in rat serum before and after AS-IV treatment, detected by ELISA. ***P<0.001 versus control. #*P*<0.05, ##*P*<0.01, and ###*P*<0.001 versus the PCOS group

AS-IV: Astragaloside IV; PCOS: Polycystic ovary syndrome; ELISA: Enzyme-linked immunosorbent assay; HOMA-IR: Homeostasis model assessment of insulin resistance

Insulin resistance is a feature of PCOS. We examined the effect of AS-IV on insulin resistance in PCOS rats. As shown in Figure 2, the level of serum insulin was higher in PCOS rats than that in control rats (compared with the Control group, *P*<0.001). The treatment of AS-IV at concentrations of 40 and 80 mg/kg significantly decreased serum level of insulin in PCOS rats (compared with the PCOS group, *P*<0.01). Also, blood glucose was increased in PCOS rats (compared with the Control group, *P*<0.001) while it was reduced after AS-IV treatment (compared with the PCOS group, *P*<0.05, *P*<0.01, and *P*<0.001). There was a significant increase in HOMA-IR in PCOS rats (compared with the Control group, *P*<0.001), which was significantly inhibited by AS-IV (compared with the PCOS group, *P*<0.05 and *P*<0.001).

***AS-IV treatment activates autophagy and PPARγ signaling in rat ovarian tissues***

**Figure 3:**
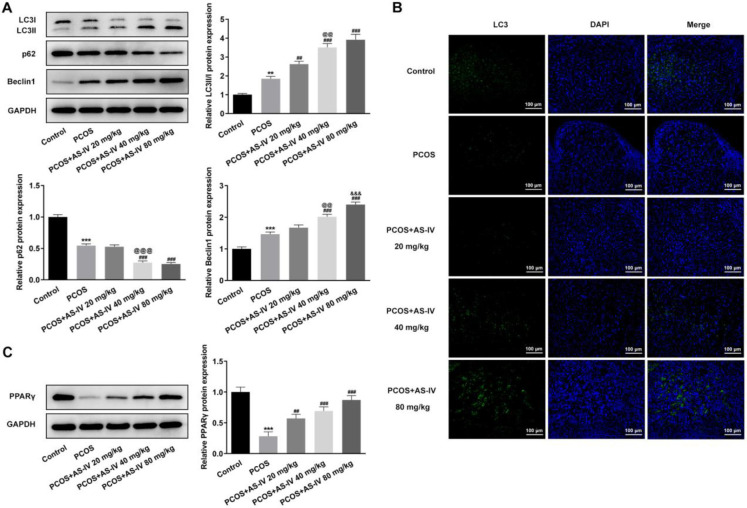
AS-IV treatment activates autophagy and PPARγ signaling in rat ovarian tissues. (A) Protein expression of autophagy-related markers LC3 II/I, p62 and Beclin1 in rat ovary tissues before and after AS-IV treatment, detected by western blot. (B) Expression of LC3 in rat ovary tissues before and after AS-IV treatment, detected by IF. (C) Protein expression of PPARγ in rat ovary tissues before and after AS-IV treatment, detected by western blot. Data are expressed as mean±SD. **P<0.01, ***P<0.001 versus control. ##*P*<0.01, ###*P*<0.001 versus the PCOS group. @@*P*<0.01, @@@*P*<0.001 versus the +AS-IV 20 mg/kg group. &&&*P*<0.001 versus the +AS-IV 40 mg/kg group

AS-IV: Astragaloside IV; PCOS: Polycystic ovary syndrome

The autophagy and PPARγ signaling-related protein expression in rat ovarian tissues after AS-IV treatment was detected by Western blot. In comparison with the controls, the protein expression levels of the autophagy markers, LC3-II/I and Beclin1, were higher and that of p62, which is associated with the inhibition of autophagy, was lower in the PCOS group (compared with the Control group, *P*<0.01 and *P*<0.001). AS-IV treatment increased the expression of LC3-II/I and Beclin1 and decreased that of p62 in the rat ovarian tissues (compared with PCOS group, *P*<0.01 and *P*<0.001; compared with +AS-IV 20 mg/kg group, *P*<0.01 and *P*<0.001; compared with +AS-IV 40 mg/kg group, *P*<0.001) (Figure 3A). Additionally, LC3 expression was decreased in the PCOS group compared with the control. Its expression was gradually increased by treatment with AS-IV, particularly at the concentration of 80 mg/kg (Figure 3B). Moreover, the protein expression levels of PPARγ were found to be low in the PCOS group (compared with the Control group, *P*<0.001), whereas AS-IV increased its expression in a dose-dependent manner (compared with +AS-IV 20 mg/kg group, *P*<0.01 and *P*<0.001) (Figure 3C). 

***AS-IV treatment activates autophagy and PPARγ signaling in KGN cells***

**Figure 4:**
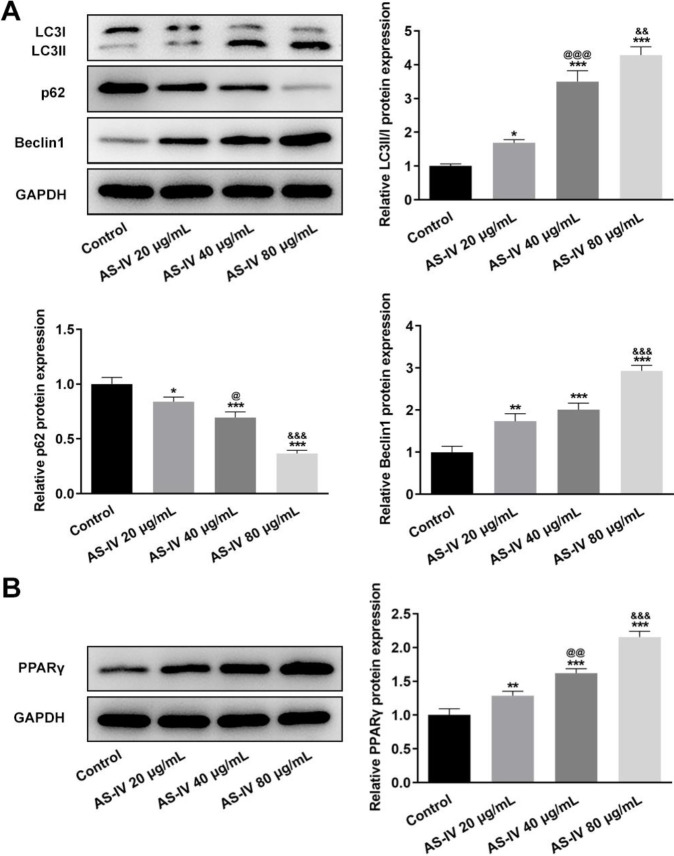
AS-IV treatment activates autophagy and PPARγ signaling in KGN cells. (A) Protein expression of autophagy-related markers LC3 II/I, p62, and Beclin1 in KGN cells before and after AS-IV treatment, detected by western blot. (B) Protein expression of PPARγ in KGN cells before and after AS-IV treatment, detected by western blot. Data are expressed as mean±SD. *P<0.05, **P<0.01, and ***P<0.001 versus control. @*P*<0.05, @@*P*<0.01, and @@@*P*<0.001 versus the AS-IV 20 μg/ml group. &&*P*<0.01, &&&*P*<0.001 versus the AS-IV 40 μg/ml group

AS-IV: Astragaloside IV; PPARγ: Peroxisome proliferator-activated receptor γ ; LC3: Light chain 3

The autophagy and PPARγ signaling-related protein expression in KGN cells after AS-IV treatment was detected by Western blot. In the KGN ovarian granulosa cells, the protein expression levels of LC3-II/I and Beclin1 were also increased by AS-IV treatment in a concentration-dependent manner, while that of p62 was decreased with the increasing AS-IV concentration (compared with the Control group, *P*<0.05, *P*<0.01 and *P*<0.001; compared with AS-IV 20 μg/ml group, *P*<0.05 and *P*<0.001; compared with AS-IV 40 μg/ml group, *P*<0.01 and *P*<0.001) (Figure 4A). Moreover, treatment with AS-IV elevated the protein expression levels of PPARγ in the KGN cells in a concentration-dependent manner (compared with the Control group, *P*<0.01 and *P*<0.001; compared with AS-IV 20 μg/ml group, *P*<0.001; compared with AS-IV 40 μg/ml group, *P*<0.001) (Figure 4B). The concentration of AS-IV at 80 μg/ml was selected for use in the following experiments.

***AS-IV inhibits the proliferation of KGN cells by activating PPARγ signaling and promoting autophagy***

**Figure 5:**
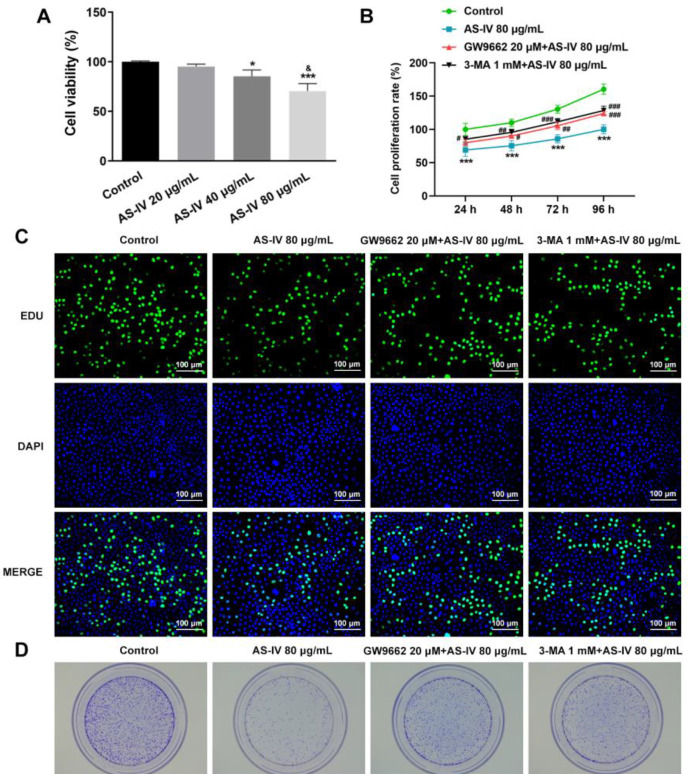
AS-IV inhibits the proliferation of KGN cells by activating PPARγ signaling and promoting autophagy (A) Viability of KGN cells treated with AS-IV was detected by CCK-8. *P<0.5 and ***P<0.001 versus control. &*P*<0.05 versus the AS-IV 40 μg/ml group. (B) Proliferation of KGN cells treated with AS-IV in the absence or presence of PPARγ inhibitor GW9662 or autophagy inhibitor 3-MA, detected by CCK-8. ***P<0.001 versus control. #*P*<0.05, ##*P*<0.01, and ###*P*<0.001 versus the AS-IV 80 μg/ml group. (C) Proliferation of KGN cells treated with AS-IV in the absence or presence of GW9662 or 3-MA observed through EdU staining. (D) KGN cell colonies following AS-IV treatment in the absence or presence of GW9662 or 3-MA. Data are expressed as mean ± SD

The proliferation and colony formation of KGN cells after AS-IV treatment were detected by CCK-8 assay, Edu staining, and colony formation assay. After KGN cells were treated with different concentrations of AS-IV, the viability of KGN cells was suppressed as the concentration increased (compared with the Control group, *P*<0.01 and *P*<0.001; compared with AS-IV 40 μg/ml group, *P*<0.05) (Figure 5A). KGN cells were pre-treated with the PPARγ inhibitor, GW9662, or the autophagy inhibitor, 3-MA, before the observation of proliferation and apoptosis. It was found that compared with the control group, AS-IV treatment inhibited KGN cell proliferation (compared with the Control group, *P*<0.001), whereas cell proliferation was augmented by pretreatment with GW9662 or 3-MA (compared with AS-IV 80 μg/ml group, *P*<0.05, *P*<0.01, and *P*<0.001) (Figure 5B). EdU staining displayed KGN cell proliferation in a more observable manner, where the AS-IV group exhibited a reduction in the number of proliferative cells in contrast to the control group; however, both GW9662 + AS-IV and 3-MA +AS-IV groups exhibited an increased number of proliferative cells (Figure 5C). Colony formation assays yielded similar results, as the number of colonies decreased in the AS-IV group and increased again following pre-treatment with GW9662 or 3-MA (Figure 5D). 

***AS-IV accelerates apoptosis of KGN cells by activating PPARγ signaling and promoting autophagy***

**Figure 6:**
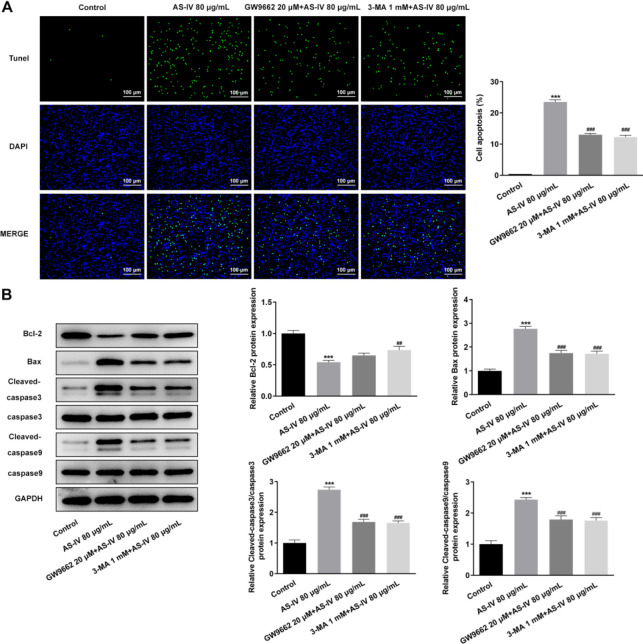
AS-IV accelerates the apoptosis of KGN cells by activating PPARγ signaling and promoting autophagy. (A) Apoptosis of KGN cells treated with AS-IV in the absence or presence of GW9662 or 3-MA, observed through TUNEL. (B) Expression of apoptosis-related proteins Bcl-2, Bax, cleaved-caspase3, and cleaved-caspase9 in KGN cells treated with AS-IV in the absence or presence of GW9662 or 3-MA, detected by western blot. Data are expressed as mean ± SD. ***P<0.001 versus control. ##*P*<0.01, ###*P*<0.001 versus the AS-IV 80 μg/ml group

The apoptosis of KGN cells after AS-IV treatment and apoptosis-related protein expression were detected by Tunel assay and Western blot. As shown in Figure 6A, the number of apoptotic KGN cells was increased by AS-IV treatment (compared with the Control group, *P*<0.001), whereas it was decreased in the presence of GW9662 and 3-MA (compared with AS-IV 80 μg/ml group, *P*<0.001). Moreover, the protein expression levels of anti-apoptotic Bcl-2 were attenuated by AS-IV treatment (compared with the Control group, *P*<0.001), which was then increased by pre-treatment with GW9662 or 3-MA (compared with AS-IV 80 μg/ml group, *P*<0.01); however, the expression of the pro-apoptotic proteins Bax, cleaved-caspase3/caspase3 and cleaved-caspase9/caspase9 exhibited the opposite effect (compared with Control group, *P*<0.001; compared with AS-IV 80 μg/ml group, *P*<0.01 and *P*<0.001) (Figure 6B). 

AS-IV: Astragaloside IV; PPARγ: Peroxisome proliferator-activated receptor γ

AS-IV: Astragaloside IV; PPARγ: Peroxisome proliferator-activated receptor γ

## Discussion

In this study, the PCOS rat model was conducted with different concentrations of AS-IV to treat the PCOS rats to see the changes in ovarian function, autophagy, and PPARγ pathway. Moreover, different concentrations of AS-IV were used to treat the KGN cells, and proliferation, apoptosis autophagy, and PPARγ pathway were analyzed. In addition, PPARγ inhibitor and autophagy inhibitor were used to reversely demonstrate the role of autophagy and PPARγ pathway in AS-IV.

AS-IV is a bioactive saponin extracted from the root of *A. membranaceus*. *A. membranaceus* is well-known in the realm of traditional Chinese medicine for its wide range of pharmacological effects. The therapeutic potential of AS-IV has also been demonstrated by substantial research on a number of diseases. Fan *et al. *(29), through their *in vivo* experiments, found that AS-IV attenuated renal dysfunction in diabetic nephropathy by suppressing eNOS acetylation and enabling phosphorylation at Ser 1177. Another study (30) reported that AS-IV regulated diabetes-associated abnormal energy metabolism and protected diabetic rats against myocardial injury through modulation of the release of PPARγ coactivator 1α and nuclear respiratory factor 1. Nejati *et al*. demonstrated that *Astragalus hamosus* could decrease levels of insulin and testosterone in PCOS rats (31). However, whether AS-IV exerts any beneficial effect on PCOS has not yet been reported, at least to the best of our knowledge. In the present study, in the animal model of PCOS, AS-IV treatment effectively attenuated the pathological injury to the ovary issues and decreased the serum levels of LH, FSH, and testosterone, as well as the LH/FSH value. An increased LH level and the LH/FSH ratio are important biomarkers for the onset of PCOS, in addition to testosterone elevation, which often signifies hyperandrogenism (32, 33). Therefore, AS-IV treatment may benefit the recovery of the ovaries from a PCOS-related pathological morphology. 

Furthermore, the present study examined the mechanism underlying the effects of AS-IV. A previous study demonstrated that AS-IV may enhance memory function and prevent cell apoptosis in AβO-challenged hippocampal neurons by activating the PPARγ/brain-derived neurotrophic factor signaling pathway (20). In another study using a rat model of PCOS, γ-linolenic acid relieved the DHEA-induced inflammatory response and down-regulated the level of leptin through up-regulation of PPARγ expression, thus improving PCOS (34). The activation of PPARγ signaling has also been found to be a link in the mechanism of action of oridonin in inhibiting the tumor growth of osteosarcoma (35). Therefore, it was hypothesized that the mechanisms responsible for the effects of AS-IV on rat ovarian tissues may be mediated by its effects on PPARγ. The results of the present study revealed that AS-IV treatment noticeably up-regulated the protein levels of PPARγ in both the rat model of PCOS and in KGN cells, suggesting that AS-IV promotes PPARγ signaling *in vitro* and* in vivo*.

Autophagy is a dual regulator in the development of PCOS: on the one hand, its inhibition has been found in theca cells of women with PCOS (36); on the other hand, androgen-activated autophagy in granulosa cells of patients with PCOS is considered to contribute to PCOS (37). A variety of extracts of *A. membranaceus*, the source of AS-IV, have exhibited perceptible effects on the regulation of autophagy (38). The findings of the present study demonstrated that the autophagy markers, LC3, LC3-II/I, and Beclin1, were up-regulated, while the level of p62, whose accumulation indicates inhibited autophagy (39), was down-regulated following AS-IV treatment in both rat ovarian tissues and KGN cells. The regulatory effects of AS-IV on autophagy were thus identified in both *in vitro* and *in vivo* models of PCOS.

The influence of AS-IV treatment on cell proliferation and apoptosis has been corroborated by extensive research. For example, AS-IV has been shown to inhibit proliferation, migration, and epithelial-mesenchymal transition of cervical cancer cells through its impact on the expression of TGF-β1 and E-cadherin (40). It has also been shown to suppress cancer cell proliferation and facilitate apoptosis and autophagy through the TGF-β/Smad signaling pathway in vulvar squamous cell carcinoma (VSCC) (41). In the present study, KGN cells exhibited a prominent decrease in proliferation and a significant increase in apoptosis following AS-IV treatment. Furthermore, studies have demonstrated that activation of the PPARγ pathway may stimulate autophagy of hepatocytes and expression of autophagy markers in mouse liver tissues, thereby improving glucose metabolism, protein homeostasis, and liver steatosis (24, 42). In a rat model of PCOS, enhanced autophagy through electroacupuncture therapy has been reported to improve insulin resistance (43). This suggests that PPARγ activation may affect proliferation and apoptosis of KGN cells by regulating autophagy. There is increasing evidence that although autophagy and apoptosis pathways are different, they are closely related. Autophagy has a protective effect on cells under certain stress conditions, which can reduce cell death by inhibiting apoptosis, but in other circumstances, autophagy is another death pathway, which can lead to increased cell death (44). Yang *et al*. demonstrated that ZnO NPs-induced autophagy promoted apoptosis in mouse spermatogonium (45). In deHP-induced apoptosis and autophagy of mouse spermatogonium, autophagy also promoted apoptosis (46). The present study revealed that the PPARγ inhibitor, GW9660, and the autophagy inhibitor, 3-MA, respectively reversed the inhibitory effects of AS-IV on cell proliferation and its promoting effect on cell apoptosis; this suggests that AS-IV-activated autophagy may inhibit proliferation and accelerate apoptosis by promoting autophagy.

## Conclusion

The present study demonstrates that AS-IV promotes autophagy by activating the PPARγ pathway to regulate the proliferation and apoptosis of the ovarian granulosa KGN cell line and improve ovarian function in rats with PCOS. The findings of the present study, based on *in vitro* and *in vivo* experiments, provide further insight into the pharmacological effects of AS-IV and suggest the possible use of AS-IV as a promising agent in the treatment of PCOS.

## Authors’ Contributions

MW and XD Designed the study, and drafted and revised the manuscript. MW, WC, and QZ Analyzed the data and searched the literature. MW, WC, QZ, and XD Performed the experiments. All authors read and approved the final manuscript.

## Availability of Data and Materials

All data generated or analyzed during this study are included in this published article.

## Ethics Approval and Consent to Participate

The animal experiment in this study was approved by the Experimental Animal Management and Ethics Committee of Zhejiang University of Traditional Chinese Medicine.

## Patient Consent for Publication

Not applicable.

## Conflicts of Interest

The authors declare that they have no competing interests.
